# The Seventh Asia Pacific Bioinformatics Conference (APBC2009)

**DOI:** 10.1186/1471-2105-10-S1-S1

**Published:** 2009-01-30

**Authors:** Michael Q Zhang, Michael S Waterman, Xuegong Zhang

**Affiliations:** 1 Cold Spring Harbor Laboratory, NY, USA; 2 Tsinghua University, Beijing, PR China; 3 University of Southern California, CA, USA

## 

The Asia Pacific Bioinformatics Conference (APBC) series, founded in 2003, is an annual international forum for exploring research, development and applications of Bioinformatics and Computational Biology. The Seventh Asia Pacific Bioinformatics Conference (APBC2009) was held at Tsinghua University, Beijing, China during January 13–16 in 2009. It brought together more than 300 researchers, professionals, industrial practitioners and students from all over the world for interaction and exchange of knowledge and ideas in broad areas of bioinformatics and computational biology. The participants came from 21 nations and regions including (in alphabetical order) Australia, Belgium, Canada, China (the Mainland, Hong Kong and Taiwan), Denmark, Finland, France, Germany, Greece, India, Japan, Korea, the Netherlands, Poland, Singapore, Switzerland, Thailand, the United Kingdom, the USA and Vietnam. This represents the largest submission and participation in the history of APBC. The conference program includes 2 keynotes (Drs. David J. Lipman and Michael S. Waterman), 5 invited talks (Drs. Michael B. Eisen, Bailin Hao, John Mattick, Pavel Pevzner and Martin Vingron), 76 selected talks, 4 tutorial sessions and 120 posters.

The titles of the keynote and invited talks are:

• David J. Lipman: *Molecular evolution and epidemiology of seasonal influenza*

• Michael S. Waterman: *Sequence analysis using Eulerian graphs*

• Michael B. Eisen: *Understanding and exploiting the evolution of Drosophila regulatory sequences*

• Bailin Hao: *Independent verification of 16S rRNA based prokaryotic phylogeny by composition vector approach*

• John Mattick: *A new understanding of the human genome*

• Pavel Pevzner:  Genome Rearrangements: from Biological Problems to Combinatorial Algorithms

• Martin Vingron: *Transcriptional regulation: computational methods, statistics, and coregulation*

The topics of the four tutorials are: *A structured approach for the dynamics modeling of biomolecular networks *(David R. Gilbert, Robin Donaldson, Monika Heiner & Rainer Breitling), *Natural language processing for biology: BioNLP *(Zhiyong Lu & Kevin Cohen), *Bayesian methods in genetic association studies *(Matthew Stephens), and *Systems biology based on integrated analysis of functional genomics data *(Olga G. Troyanskaya).

The APBC series had a tradition of emphasis on rigorous algorithm topics in bioinformatics and computation. In this year's conference, this hallmark was retained while the biological contexts of the topics were given more emphasis. Authors from more diverse backgrounds of computer science, biology, statistics, electrical engineering, medical sciences, chemistry and physics contributed to the conference. The selected papers and posters cover a wide range of themes in current bioinformatics and computational biology. The biological questions studied can be categorized into the following major fields:

• DNA sequence analysis, alignment, evolution and comparative genomics, sequence assembly especially in the context of second-generation deep sequencing technology.

• Gene regulation and expression analysis, including microarray data analysis and integration, transcriptional regulation, alternative splicing, and epigenomic factors.

• RNA structure and function, especially non-coding RNAs including microRNAs and RNAi.

• Proteins and proteomics, including protein structure, subcellular location and function and mass spectrometry data processing.

• Biological pathways, networks and systems biology.

• Disease and medical application study, genome-wide association studies.

A total of 204 submissions were received and reviewed by a strong 63-member international Program Committee so that each submission is reviewed at least by 2 PC members, and more than 30 additional reviewers were invited by the PC members to provide extra reviews. The selected talks (all but one are collected in this supplement to *BMC Bioinformatics*) represent a 37% acceptance rate, and the authors have carefully revised the papers according to the reviews before the publication. A full proceedings of APBC2009, including all the accepted full papers and abstracts, is published by Tsinghua University Press.

In conclusion we express our deep appreciation to the PC members and the additional reviewers who worked on a very tight schedule, sharing their valuable time and exquisite expertise in support of the review process. The PC members are:

• Tatsuya Akutsu, Kyoto University

• Joel Bader, Johns Hopkins University

• Harmen Bussemaker, Columbia University

• Andrea Califano, Columbia University

• Francis Y.L. Chin, Hong Kong University, Hong Kong

• Phoebe Chen, Deakin University

• Runsheng Chen, Institute of Biophysics, CAS

• Xue-wen Chen, University of Kansas

• Nadia El-Mabrouk, University of Montreal

• Mikhail Gelfand, Russian Academy of Science

• Roderic Guigo, Centre de Regulacio Genomica, Barcelona

• Jing-Dong Jackie Han, CA

• Jenn-Kang Hwang, National Chiao Tung University

• Sridhar Hannenhalli, University of Pennsylvania

• Daniel Huson, University of Tübingen

• Rui Jiang, Tsinghua University

• Tao Jiang, University of California Riverside

• Samuel Kaski, Helsinki University of Technology

• Uri Keich, Cornell University

• Luhua Lai, Peking University

• Tak Wah Lam, University of Hong Kong

• Charles Lawrence, Brown University

• Chris Lee, UCLA

• Doheon Lee, KAIST

• Sang Yup Lee, KAIST

• Cheng Li, DFCI/HSPH

• Hao Li, UCSF

• Jinyan Li, Nanyang Technological University

• Wen-Hsiung Li, University of Chicago

• Wentian Li, Feinstein Institute for Medical Research

• Yixue Li, CAS and Shanghai Jiaotong University

• Jun S. Liu, Harvard University

• Jingchu Luo, Peking University

• Bin Ma, University of Western Ontario

• Hiroshi Mamitsuka, Kyoto University

• Satoru Miyano, University of Tokyo

• Kenta Nakai, University of Tokyo

• Jurg Ott, Beijing Institute of Genomics, CAS

• Laxmi Parida, IBM Watson Research Center

• John Quackenbush, Harvard School of Public Health

• Nikolaus Rajewsky, MDC-Berlin

• Ron Shamir, Tel Aviv University

• Steven Skiena, State University of New York at Stony Brook

• Yun S. Song, University of California at Berkeley

• Paul Spellman, Lawrence Berkeley National Laboratory

• Fengzhu Sun, University of Southern California

• Zhirong Sun, Tsinghua University

• Masaru Tomita, Keio University

• Martin Vingron, Max Planck Institute for Molecular Genetics

• Lusheng Wang, City University of Hong Kong

• Michael S. Waterman, University of Southern California

• Limsoon Wong, National University of Singapore

• Wing H. Wong, Stanford University

• Eric Xing, Carnegie Mellon University

• Ying Xu, University of Georgia, USA

• Hong Xue, Hong Kong University of Science and Technology

• Hong Yan, City University of Hong Kong

• Weichuan Yu, Hong Kong University of Science and Technology

• Luoxin Zhang, National University of Singapore

• Michael Q. Zhang, Cold Spring Harbor Laboratory

• Xuegong Zhang, Tsinghua University

• Hongyu Zhao, Yale University

• Qing Zhou, University of California at Los Angeles

We would also like to thank all local Organizing Committee members for their hard work behind the scenes, especially the Poster Co-chairs Shao Li and Xiaoyan Zhu. We also wish to thank Eric P. Xing and Rui Jiang for organizing the excellent Tutorial session. We thank the conference secretaries Victor Renault, Xi Ma, Haiyan Luo, Tingting Li, Shicai Fan and Wendian Yan for their hard work on the website, technical editing, registration and many other miscellaneous tasks. We thank Tao Jiang, Phoebe Chen, Limsoon Wong and Jingchu Luo for their help and numerous suggestions. And we thank the National Natural Science Foundation of China (NSFC), the National Basic Research Program of China, the National Hi-tech Research and Development Program of China and Tsinghua University for their support.

## Competing interests

The authors declare that they have no competing interests.

